# A285 CHANGE IN ALT DURING MODIFIED OPTIFAST WEIGHT LOSS PROGRAM IN INDIVIDUALS AT RISK FOR NON-ALCOHOLIC FATTY LIVER DISEASE

**DOI:** 10.1093/jcag/gwac036.285

**Published:** 2023-03-07

**Authors:** L Meddings Maybury, E Kelly, R Dent, B Bielawska

**Affiliations:** 1 The Ottawa Hospital, Ottawa, Canada; 2 Gastroenterology, The Ottawa Hospital; 3 Ottawa Hospital Research Institute, Ottawa, Canada

## Abstract

**Background:**

Obesity is linked to various health complications, including diabetes, metabolic syndrome, cardiovascular disease, and non-alcoholic fatty liver disease (NAFLD). NAFLD is the most frequent cause of abnormally high alanine aminotransferase (ALT) levels. ALT is released at higher levels during hepatocellular injury and represents a simple, low cost and non-invasive method of assessing liver damage. There are conflicting data on ALT response to weight loss between men and women, with some evidence of transient increase in ALT levels in women.

**Purpose:**

The aim of this study was to assess the impact of a dietary weight loss intervention with Optifast on ALT in patients with obesity who have baseline elevated ALT levels.

**Method:**

This was a retrospective cohort study of Ontario adults who participated in a 26 week weight loss program, including 6 or 12 weeks of low-calorie liquid diet meal replacement with Optifast 900^®^ (Nestlé, Canada), between 1992 and 2015. We included patients with elevated baseline ALT levels, defined by > 40 U/L, who had at least one follow-up ALT between week 15 and 26. We excluded patients with nonadherence to dietary intervention, established liver disease or using hepatotoxic drugs, excessive alcohol intake (>11U/wk for females and >14U/wk for males), and insufficient data to calculate FIB-4. The primary outcome was change in ALT levels, measured by normalization (post-intervention ALT < 40 U/L), percentage decrease and absolute decrease. Multiple linear regressions were obtained for the relationship between ALT changes and age, sex, body mass index (BMI) and FIB-4. All analyses were conducted in SPSS. *P* value of ≤0.05 was considered statistically significant.

**Result(s):**

444 patients met study criteria. Mean age was 47.1 years +/- 10.9, 49 % of patients were female, and mean BMI was 43.5 kg/m^2^ +/- 7.9. All patients lost weight, with mean weight loss 27.3 kg +/- 11.5 and a mean 20.7% decrease from baseline weight. Mean ALT at baseline was 58.9 U/L +/- 25.4 and mean ALT post intervention was 32.3 U/L +/- 28.1 (p <0.01). 85.1% of patients had normalization of ALT. Mean % decrease in ALT was 41.9% and mean absolute decrease in ALT was 26.6 U/L +/- 25.4. Younger age and higher baseline FIB-4 were significantly associated with a larger decrease in ALT levels, while sex and initial BMI were not significantly associated with changes in ALT.

**Conclusion(s):**

In patients with obesity and baseline elevated ALT, dietary weight loss intervention with Optifast leads to ALT normalization in most patients, regardless of sex. Younger age at baseline and higher baseline FIB-4 are significantly associated with greater decrease in ALT.

**Please acknowledge all funding agencies by checking the applicable boxes below:**

None

**Disclosure of Interest:**

None Declared

